# Supplementary material for: The memory of airway epithelium damage in smokers and COPD patients
Source: Life Sci Alliance. 2023 Dec 29;7(3):e202302341. doi: 10.26508/lsa.202302341 (PMC10756916; doi:10.26508/lsa.202302341)
Supplement: Supplementary file 1 [file LSA-2023-02341_TableS1.docx]

|  | **Non-smoker controls**  **(n=5)** | **Smoker controls**  **(n=8)** | **COPD1-2**  **(n=6)** | **COPD3-4**  **(n=6)** |  |
| --- | --- | --- | --- | --- | --- |
| **N (Male/Female)** | 5 (2/3) | 8 (5/3) | 6 (2/4) | 6 (3/3) | ns |
| **Age** | 69.4 ± 14.1 | 62.4 ± 7.1 | 62.0 ± 4.6 | 61.7 ± 2.1 | ns |
| **Smoking history**  **(never/former/current n)** | 5/0/0 | 0/5/3 | 0/3/3 | 0/6/0 | p<0.01 |
| **Pack-years** | NA | 37.0 ± 27.1 | 42.7 ± 24.6 | 53.3 ± 30.3 | ns |
| **If applicable, duration since smoking cessation (months)** | NA | 275.4 ± 149.7 | 85.6 ± 56.9 | 108.1 ± 116.5 | ns |
| **FEV1 (% of PV)** | 110.8 ± 18.5 | 99.1 ± 9.6 | 70.3 ± 8.0*^#^ | 26.7 ± 7.8*^#¶^ | p<0.0001 |
| **FEV1/VC ratio (%P)** | 79.7 ± 8.7 | 74.3 ± 3.3 | 66.0 ± 7.9*^#^ | 35.2 ± 7.5*^#¶^ | p<0.0001 |
| **DLCO (% of PV)** | 92.8 ± 9.9 | 88.5 ± 10.7 | 64.2 ± 15.3 | 36.5 ± 8.3*^#^ | p<0.01 |
| **BMI (kg.m^-2^)** | 26.7 ± 7.6 | 26.7 ± 5.3 | 27.5 ± 6.2 | 25.4 ± 4.2 | ns |
| **Inhaled corticosteroids (n/total N)** | 0/5 | 0/8 | 1/6 | 5/6 | p<0.001 |
| **Surgical indication**   - **Neoplasia**    - **SCC**   - **AC**   - **Carcinoid tumour**   - **Pulmonary metastasis of other cancers** - **Lung transplant** - **Other** - **Declined lung donor** | 4/5  0  2  2  0  0  0  1 | 8/8  2  4  1  1  0  0  0 | 5/6  1  3  0  1  0  1  0 | 0/6  0  0  0  0  6  0  0 | p<0.0001^1^ |
| **Table S1 \| Patient series for short-term (5 weeks) ALI culture with/without inflammatory condition.** Data are presented as mean ± SD, unless otherwise stated. Demographic data, lung function tests, smoking history and inhaled corticotherapy are stated for the patient groups, classified according to smoking history and the presence and severity of airflow limitation. AC, adenocarcinoma; ALI, air/liquid interface; BMI, body mass index; COPD, chronic obstructive pulmonary disease; DLCO, diffusing capacity of the lung for CO; FEV1, forced expiratory volume in 1 s; FVC, forced vital capacity; NA, not applicable; PV, predicted values; SCC, squamous cell cancer.  * = p<0.05 compared to non-smoker controls.  ^#^ = p<0.05 compared to smoker controls  ^¶^ = p<0.05 compared to COPD1-2 patients  ns, not significant  ^1^, comparison between “Neoplasia”, “Lung transplant” and “Other” groups. | | | | | |
