# Supplementary material for: The memory of airway epithelium damage in smokers and COPD patients
Source: Life Sci Alliance. 2023 Dec 29;7(3):e202302341. doi: 10.26508/lsa.202302341 (PMC10756916; doi:10.26508/lsa.202302341)
Supplement: Supplementary file 2 [file LSA-2023-02341_TableS2.docx]

| **Gene** | **FORWARD primer (5’-3’)** | **REVERSE primer (3’-5’)** | **Amplicon size (bp)** | **Melting t° (°C)** |
| --- | --- | --- | --- | --- |
| **Housekeeping genes** | | | | |
| *RPL27* | TGG TAG GGC CGG GTG GTT GC | ACT TTG CGG GGG TAG CGG TC | 185 | 60 |
| *RPS13* | TCG GCT TTA CCC TAT CGA CGC AG | ACG TAC TTG TGC AAC ACC ATG TGA | 153 | 60 |
| *RPS18* | TGT GGG CCG AAG ATA TGC T | TGA TCA CAC GTT CCA CCT CAT | 101 | 60 |
| **Target genes** | | | | |
| *CDH1* | GCT GGA CCG AGA GAG TTT CC | CGA CGT TAG CCT CGT TCT CA | 179 | 60 |
| *CXCL8* | CTC TGT GTG AAG GTG CAG TTT TG | AAC TTC TCC ACA ACC CTC TGC | 223 | 60 |
| *DNAI1* | GAG TTG ACC GAT GCG GAG TT | GGT TCC CAA CCT GGG TGT AG | 163 | 60 |
| *DNAI2* | GCG ATT CAT ACA TCT GGG AC | CAG CAG GCT ATC TGT CCA T | 145 | 60 |
| *FOXA3* | TGC TGG GCT CAG TGA AGA TG | GTC ATG TAG GAG TTG AGG GGG | 124 | 60 |
| *FOXJ1* | CCT GGC AGA ATT CAA TCC G | GCG TAC TGG GGG TCA AT | 115 | 60 |
| *IL6* | AGT TCC TGC AGA AAA AGG CAA AG | TGA GGT GCC CAT GCT ACA TTT | 198 | 60 |
| *MCIDAS* | GAC GCG CTT GTT GAG AAT AA | CAC GTT CCG CTC CTT GAG | 84 | 60 |
| *MYB* | TAC TGC CTG GAC GAA CTG ATA A | CTG GCT GGC TGG CTT TTG AA | 111 | 60 |
| *PIGR* | CTC TCT GGA GGA CCA CCG T | CAG CCG TGA CAT TCC CTG | 78 | 60 |
| *SNAI1* | CGA GTG GTT CTT CTG CGT TA | CTG CTG GAA GGT AAA CTC TGG A | 157 | 60 |
| *SNAI2* | ACT GGA CAC ACA TAC AGT GAT T | ACT CAC TCG CCC CAA AGA TG | 199 | 60 |
| *SPDEF* | TGA CCT TGG AGG AGC ACT CG | CAT GGG ATC TGC GGT GAT GTT | 110 | 60 |
| *TJP1* | GTG GTT CTT CGA GAA GCT GGA | TGC AGG CGA ATA ATG CCA GA | 170 | 60 |
| *TWIST1* | CCG GAG ACC TAG ATG TCA TTG T | CCC ACG CCC TGT TTC TTT GA | 150 | 60 |
| *VIM* | CGG GAG AAA TTG CAG GAG GA | AAG GTC AAG ACG TGC CAG AG | 105 | 60 |
| *ZEB2* | CAC AAG CCA GGG ACA GAT CA | TCG TAA GGT TTT TCA CCA CTG T | 146 | 60 |
| **Table S2 \|** List of the primers used for RT-qPCR. | | | | |
