# Supplementary material for: The memory of airway epithelium damage in smokers and COPD patients
Source: Life Sci Alliance. 2023 Dec 29;7(3):e202302341. doi: 10.26508/lsa.202302341 (PMC10756916; doi:10.26508/lsa.202302341)
Supplement: Supplementary file 3 [file LSA-2023-02341_TableS3.docx]

| **Target** | **Species** | **Brand and reference** |
| --- | --- | --- |
| **Primary antibodies** | | |
| *E-cadherin* | Mouse monoclonal antibody | Dako M3612 |
| *GAPDH* | Rabbit polyclonal antibody | Sigma G9545 |
| *pIgR* | Rabbit polyclonal antibody | Home made |
| *Occludin* | Rabbit polyclonal antibody | Merck-Millipore ABT146 |
| *Vimentin* | Mouse monoclonal antibody | Dako M0725 |
| **Secondary antibodies** | | |
| *Anti-Rabbit HRP-conjugated* | Goat polyclonal antibody | Cell signalling 7074S |
| *Anti-mouse HRP-conjugated* | Sheep polyclonal antibody | Sigma A6782 |
| **Table S3 \|** List of the primary and secondary antibodies used for western blot. | | |
