# Supplementary material for: The memory of airway epithelium damage in smokers and COPD patients
Source: Life Sci Alliance. 2023 Dec 29;7(3):e202302341. doi: 10.26508/lsa.202302341 (PMC10756916; doi:10.26508/lsa.202302341)
Supplement: Supplementary file 4 [file LSA-2023-02341_TableS4.docx]

| **Target** | **Species** | **Brand and reference** |
| --- | --- | --- |
| **Primary antibodies** | | |
| *bêta-tubulin IV* | Mouse monoclonal antibody | Sigma T7941 |
| *MUC5AC* | Mouse monoclonal antibody | Acris AM50143PU-N |
| *pIgR* | Rabbit polyclonal antibody | Home made |
| *Mouse IgG_1_ Isotype* | Mouse monoclonal antibody | eBiosciences 14-4714-82 |
| *Rabbit IgG Isotype* | Rabbit polyclonal antibody | Homemade antibody |
| **Secondary antibodies** | | |
| *Anti-Rabbit HRP-conjugated* | Goat polyclonal antibody | Cell signalling 7074S |
| *Anti-mouse HRP-conjugated* | Sheep polyclonal antibody | Sigma A6782 |
| **Table S4 \|** List of the primary and secondary antibodies used for tyramide signal amplification-enhanced immunofluorescence staining. | | |
